# Direct oral anticoagulant use in left ventricular thrombus

**DOI:** 10.1186/s12959-020-00242-x

**Published:** 2020-10-29

**Authors:** Zafar Ali, Nicholas Isom, Tarun Dalia, Farhad Sami, Uzair Mahmood, Zubair Shah, Kamal Gupta

**Affiliations:** University of Kansas Medical Center, 3901 Rainbow Blvd, Kansas City, KS66160 USA

**Keywords:** Left ventricular thrombus, Warfarin, Direct oral anticoagulant, Cardiomyopathy

## Abstract

Left ventricular thrombus (LVT) is associated with a significant risk of ischemic stroke (IS) and peripheral embolization. Societal guidelines recommend the use of warfarin, with direct oral anticoagulants (DOACs) only for patients unable to tolerate warfarin. We studied the natural history of LVT with anticoagulation (AC) with emphasis on comparing warfarin and DOAC use. In this single center study, we identified patients with a confirmed LVT. Type and duration of anticoagulation, INR levels and clinical outcomes (bleeding, ischemic stroke or peripheral embolization, and thrombus resolution) were recorded. LVT was confirmed in a total of 110 patients. Mean age was 59 + 14 years. 79% were men. Underlying etiology was chronic ischemic cardiomyopathy in 58%, non-ischemic cardiomyopathy in 23%. AC was started in 96 (87%) patients. At 1 year follow up, 11 patients (10%) had a stroke while on any AC (2 had hemorrhagic stroke and 9 had IS). Of those with IS, 7 were on warfarin (71% of those had subtherapeutic INR) and 2 patients on DOACs had IS. The 1-year risk of any stroke was 15% in warfarin group (12% risk of ischemic stroke) compared to 6% in the DOACs group (*p* = 0.33). 37 (63%) patients on warfarin and 18 (53%) on DOACs had resolution of thrombus (*p* = 0.85). One-year risk of stroke with LVT is high (10%) even with AC. Most patients IS on warfarin had subtherapeutic INR. There was no statistical difference in stroke risk or rate of thrombus resolution between warfarin and DOACs treated patients.

Left ventricular thrombus (LVT) mostly occurs in patients with significant systolic dysfunction and can have devastating consequences from ischemic stroke (IS) and peripheral embolism. Risk factors associated with LVT formation are large anterior myocardial infarction, LV systolic dysfunction and severe wall motion abnormalities [1]. The incidence of LVT in the pre-percutaneous coronary intervention (PCI) era was reported to be as high as 40% but has decreased significantly (about 4%) in the primary PCI era [1, 2]. Various societal guidelines recommend 3 to 6 months of anticoagulation (AC) with warfarin (or up till thrombus resolution) [3–5]. However, there is a lack of good evidence to guide these recommendations. All guidelines recommend using vitamin K antagonists. The 2014 stroke guidelines do suggest that direct oral anticoagulants (DAOCs) could be used in patients with warfarin intolerance, though there is very little data to support this use [4]. The goal of this study was to evaluate the natural history of LVT and incidence of thrombus resolution, with special emphasis on comparing warfarin and DOACs.

In this single center, retrospective study, we included all patients with confirmed LV thrombus. The study was approved by the institutional review board. Using natural language processing algorithms, we identified patients who had LVT in cardiac imaging reports. Then manual review of the electronic medical records was done to exclude patients who did not actually have a confirmed LVT. A detailed chart review was done in these patients to record the demographic details, clinical history, imaging and laboratory data and outcomes on follow up.

A total of 110 patients were confirmed to have LVT. The initial imaging modality that diagnosed the LVT was transthoracic echocardiography (TTE) in 102 patients, cardiac catheterization in 4 patients, computed tomography angiography in 2 patients and magnetic resonance imaging in 2 patients. Mean age was 59 ± 14 years. 79% of the patients were men, 66.4% were Caucasian and 19% were African American. Diabetes was present in 66.6 and 26.3% had atrial fibrillation. Underlying etiology was chronic ischemic cardiomyopathy in 58%, non-ischemic cardiomyopathy in 23%, acute MI in 15% and Takotsubu cardiomyopathy in 3% patients.

AC was started in 96 (87%) of the patients with the remaining deemed to have significant contraindication to anticoagulation by the treating physicians or per patient choice. Of those started on AC, 60 patients (63%) were started on warfarin, 32 patients (33%) on DOACs (18 on Rivaroxaban, 13 on Apixaban and 1 on Dabigatran) and 4 patients (3%) on long-term enoxaparin. Of those patients started on AC, 48 were started on heparin initially, 16 on Enoxaparin, 5 were not bridged and information was not available on the remaining 25 regarding bridging. There were no significant differences in demographics and underlying comorbidities between those on warfarin and those on DOACs, except that peripheral artery disease was significantly more frequent in warfarin group (Table 1). Concurrent antiplatelet therapy was with aspirin in 65.45%, clopidogrel in 14.55%, ticagrelor in 0.91%, prasugrel in 1.82%.

**Table 1:** Baseline characteristics of patients with final anticoagulation of warfarin and DOACs

	Warfarin (*N* = 60)	DOAC (32)	*P*-value
Age	58.0 (SD 16.3)	59.2 (SD 11.9)	0.71
BMI	28.5 (SD 6.2)	29.2 (SD 4.7)	0.57
Race			0.33
Caucasian	38	25	
African Americans	14	5	
Others/Unknown or More than one	8	2	
Gender			0.96
Male	49	26	
Female	11	6	
Smoker			0.11
Current smoker	7	7	
Former smoker	32	10	
Never smoker	21	15	
Congestive heart failure	45	25	0.74
Mean ejection fraction at the time of diagnosis	23.2% (SD 11.2)	23.0% (SD 9.4)	0.95
Diabetes mellitus	18	12	0.47
Atrial fibrillation	18	9	0.85
Ventricular tachycardia	11	4	0.47
PAD	14	2	0.04
Abdominal aortic aneurysm	5	1	0.34

Follow up information was available for 84.6% of patients of whom 24.6% had a follow up of less than a year, 22.7% of 1–3 years, 17.3% for 3–5 years, and 18.2% had a follow up of greater than 5 years.

At one-year follow up, 11 patients (11.4%) had had a stroke while on AC. Two of these patients had hemorrhagic stroke (both on warfarin) and 9 patients had an IS. Of those with IS, 7 were on warfarin and 2 patients on DOACs. Of those with ischemic stroke on warfarin, 71% had sub-therapeutic INR. The 1-year risk of any stroke was 15% in warfarin group (12% risk of IS) compared to 6% in the DOACs group (*p* = 0.33). Five patients (5.5%) had peripheral embolism while on AC (all on warfarin). Thus, the incidence of any stroke or systemic embolism in the warfarin group was 26.6% (16 patients) compared to 6% (2 patients) with DOACs (*p =* 0.0001)*.*

A total of 55 patients (53%) were found to have resolution of thrombi as shown in Fig. 1. Overall 37 (62%) patients on warfarin and 18 (53%) on DOACs had resolution of thrombus (*p* = 0.85) while on anticoagulation. We further found there were significant differences in the temporality of thrombus resolution between warfarin and DOACs use with significantly higher percentage resolving within the first month with DOACs compared to warfarin (34% vs 12%, *p* = < 0.00001) (Fig. 1). It is possible that earlier resolution of thrombus accounts for the overall lower risk of thromboembolic events seen with DOACs over warfarin even though overall thrombus resolution rates were similar.

**Fig. 1:**
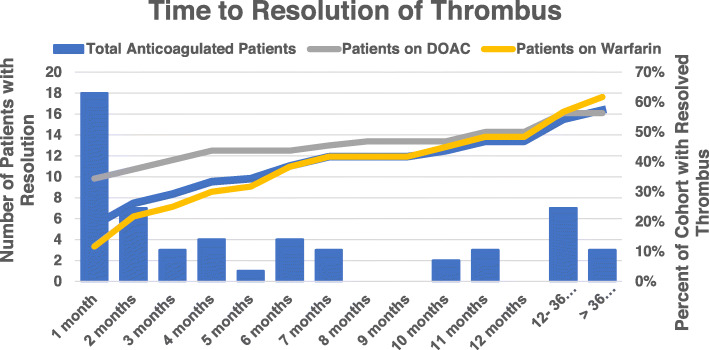
Time to resolution of thrombus on patients with warnfarin vs DOACs

There is paucity of literature on the use of DOACs in LVT. In a recent well-conducted single center study with 157 patients, the authors reported a stroke/ embolism rate of 22.2% over a median follow up of 632 days [6]. In this study, 77 patients were on vitamin K antagonists (most were on fluindione) and 36 were on DOACs. The thrombus resolution rate was 62.3% at a median time of 103 days. The authors do not report data on thrombus resolution (incidence or temporality) or risk of adverse events stratified by AC used but do mention that there was no statistical difference in rates of major adverse cardiac events or thrombus resolution between DOACs and Vitamin K antagonists. We found no other study that has assessed type of AC and outcomes in LVT.

In conclusion, we found a significant 1-year risk of stroke or systemic embolism in patients with LVT even while on anticoagulation. The thrombus resolution rates were similar between DOACs and warfarin, though resolution occurred temporally significantly earlier with DOACs. The combined endpoint of any stroke of peripheral embolism was also significantly lower in the DOACs group. Our study provides preliminary evidence that DOACs use is safe and effective in LVT and given lack of larger study, these findings could help guide treatment in these patients.

## Data Availability

The dataset used during the study are available from the corresponding author on reasonable request.

## Abbreviations

LVT: Left Ventricular Thrombus
IS: Ischemic Stroke
PCI: Percutaneous coronary intervention
AC: Anticoagulation
DOACs: Direct Oral Anticoagulants
TTE: Transthoracic echocardiography
